# The influence of the multifactorial falls prevention programme on mortality

**DOI:** 10.1177/14034948241285559

**Published:** 2024-12-05

**Authors:** Niko Korpi, Marja Mikkelsson, Tomi Korpi, Hannu Kautiainen

**Affiliations:** 1 Doctoral Programme in Clinical Research University of Helsinki, Finland; 2 Orimattila Health Centre, Wellbeing Services County of Päijät-Häme, Orimattila, Finland; 3 Faculty of Medicine, University of Helsinki, Finland; 4 Department of Rehabilitation, Wellbeing Services County of Päijät-Häme, Lahti, Finland; 5 The Hospital District of Helsinki and Uusimaa, Helsinki, Finland; 6 Folkhälsan Research Centre, Helsinki, Finland; 7 Unit of Primary Health Care, Kuopio University Hospital, Finland

**Keywords:** Adult, mortality, accidental falls, accidental injuries, osteoporosis, risk assessment, accident prevention, patient care team, retrospective studies, cohort studies

## Abstract

**Aims:**

Multifactorial falls prevention programmes (MFFPs) can prevent falls and fall-related injuries. We aimed to study MFFP patients’ mortality compared with their sex-, age- and residence-matched population-based controls.

**Methods:**

This study is a Finnish single-centre retrospective register-based controlled cohort study of a total of 527 home-dwelling MFFP patients and their 3:1 age-, sex- and residence-matched population-based controls (*n* = 1581), who had not attended the MFFP.

**Results:**

During the follow-up, the cumulative mortality of all patients was 40.4, and of controls 39.1 %. Hazard ratio was 0.82 (95% confidence interval 0.68 to 0.99), *p*= 0.041. Case patients had a 2.7 times greater risk to die due to accidents, but they had a lower risk to die due to dementia, compared with the control group. The 72-years-old or older participants had a lower mortality rate than the controls during follow-up.

**Conclusions:**

**The MFFP seems to relate to a lower all-cause mortality when comparing MFFP patients with their age-, sex- and residence-matched controls. However, the MFFP did not seem to relate to a lower injury-related mortality. The relationship between the MFFP and lower all-cause mortality seemed to be strongest in the patients aged 72 years or older. Due to the study setting and population-based control group, it is difficult to draw solid conclusions and further studies are needed. A randomized controlled trial comparing the MFFP with standard care would give better insight on the effectiveness of a MFFP on mortality.**

## Background

The increasing number of older adults face numerous health-related risks and a decline in functional abilities that induce falls and related injuries which can lead to hospitalization, accident-related surgery, institutionalization and eventually premature mortality [1–3]. In Finland, injuries and intoxications are the second biggest cause of treatment periods in specialized tertiary health care and third in the health centre ward treatment periods [4]. Likewise, accidents are the fourth most common cause of death of the Finnish people. This means roughly 2600 yearly casualties [4]. Most of the accidents happen in spare time and in the home environment. The most common accident is falling [4,5]. Kannus et al. discovered that the adult women’s rate (per 100,000 persons) of fall-induced deaths in Finland has increased from 16 in 1971 to 23 in 2016 [5]. In men, this rate rose from 14 in 1971 to 29 in 2016 [5]. However, this elevation of fall-induced deaths is not seen among 50 years or older Finns whose fall-related deaths have started to decline in both sexes from the year 2005 [6].

Finnish older adults suffer from a growing incidence of the fall-induced injuries [7,8]. Greater incidence of these unwanted outcomes will be seen with the ageing population in the near future if effective countermeasures are not established. The need for falls risk screening and prevention interventions has already been acknowledged [1–3] and found somewhat effective [2,9–15] even though some intervention programmes were unable to prevent adverse outcomes [16,17]. Potential risk factors for falling and to target an intervention for the older people include polypharmacy [13], physical inactivity [11], sarcopenia [18], slow gait speed [19] and cognitive frailty [20]. Chronic diseases make it harder to maintain physical activity and a physically inactive person has a risk to become chronically ill. An increase in physical activity is a way to prevent the premature deaths of the chronically ill [21].

The multifactorial falls prevention programme (MFFP) in Lahti, the capital city of Päijät-Häme region, was based on a successful Finnish MFFP that had been able to prevent falls and fall-related injuries in the cities of Lappeenranta and Tampere [12].

This study aimed to investigate retrospectively MFFP patients’ mortality compared with their sex-, age- and residence-matched population-based controls.

## Methods

### Design

This study was a single-centre retrospective register-based controlled cohort study of a total of 527 MFFP patients, and all of them were included in the estimations. The MFFP was conducted in the years between 2010 and 2015. The MFFP patients were independently dwelling MFFP patients residing in the city of Lahti, Finland.

During the study period, patients with an increased risk for osteoporosis, falling and fall-related injuries were identified and treated by the local public health care professionals in Lahti. The standard falls prevention and osteoporosis care in Lahti was led by nurses specially trained in osteoporosis and falls prevention in the local health care centres. The patients were referred to the MFFP if they had multimorbidity and difficulties in functioning [22].

The whole population of the city was not screened and the MFFP’s capacity was limited. This study used baseline data of the study group collected from the patient registry system (Pegasos) which was used by the MFFP health care professionals. The data included patient records from the MFFP. All information was collected by a study assistant in May 2019. Baseline information of the study group was obtained at the data collection.

The control group (*n* = 1581) was formed retrospectively by tracking three population-based controls per one patient (3:1) of the same age, sex and residence (Lahti). The controls had not attended the MFFP and were part of the same population. Both groups belonged to the same health care system and their health care needs would have been taken care of by the same health care provider. The controls were searched for in October 2020 by the Digital and Population Data Services Agency. The outcome of interest was mortality including diagnosis-based mortality. The baseline was defined as the date of the first visit to the MFFP. Data on mortality was obtained in May 2021 from the national statistical authority, Central Statistical Office of Finland. Causes of death were classified according to the International Classification of Diseases and Related Health Problems, 10th Revision.

The study was approved by the respective patient registry holder of Päijät-Häme Central Hospital. No ethics committee approval was required as no patients were contacted or identifiable by the authors.

### The MFFP

The MFFP for the 527 patients started with two appointments with a physiotherapist. After that, the patient was referred to the general practitioner (GP) either as an appointment or by consultation. The health professionals had received training to interview and test patients. All of the MFFP patients completed these appointments and, thus, were included in the mortality analyses. The MFFP focused on both primary and secondary falls prevention [22].

The first appointment included an interview, tests and measurements. Self-reported health (SRH), Mikkeli Osteoporosis Index [23], weight, height, body mass index (BMI) and waistline were measured. The patient reported their use of tobacco and alcohol, hearing ability, sight, memory, mood, sleep, nutrition and incontinence. The patient was given a self-help guide named *Remaining years* (in Finnish, *Turvallisia vuosia*) [24] at the end of the appointment. Then the patient was directed to osteoporosis blood work and bone density imaging if needed [22].

The second appointment included an evaluation of the patient’s functional ability, for example, the ability to walk and sustain balance. After the evaluation, the patient was given an individual physical exercise plan which might have included a referral to group exercise activities or a rehabilitation group led by a physiotherapist, if needed. After this, the GP interviewed the patient. The patient’s potential risks of medication problems, osteoporosis, fractures, falls, symptoms and diseases were evaluated. After the anamnesis, the GP did a comprehensive clinical status, which was complemented with an evaluation of the previously taken blood work. After the examination, a treatment plan was contrived to focus on the recent findings, including possible osteoporosis medication and deprescribing of potentially hazardous medication [22].

Most of the patients had a follow-up which included a clinical evaluation of their individual rehabilitation efforts. Further adjustments were made to the treatments and training regime, if needed. One hundred and six (20%) participants had a follow-up after six months, and 337 (64%) patients had a follow-up at 12 months. The follow-ups were not part of the main MFFP and had only clinical relevance.

### Statistical analyses

The descriptive statistics are presented as means with standard deviations (SDs) or counts with percentages. The Kaplan–Meier method was used to estimate the cumulative mortality. We used the Cox proportional hazards model to calculate the crude and adjusted hazard ratios for death. A possible non-linear relationship between the age and the hazard of death was assessed by using three-knot-restricted cubic spline Cox regression model. The length of the distribution of knots was located at the 10th, 50th and 90th percentiles; knot locations were based on Harrell’s recommended percentiles [25]. Fine and Gray competing risks regression models were used to calculate subhazard ratios (sHRs) for different causes of death. For each cause of death, the rest of the causes were considered as competing risks. The ratio of observed to expected number of deaths, the standardized mortality ratio (SMR) for all-cause deaths, was calculated using subject-years methods with 95% confidence intervals (CIs). The expected number of deaths was calculated based on sex-, age- and calendar-period-specific mortality rates in the Finnish population (Official Statistics of Finland). Ninety-five per cent CIs were computed by assuming that the observed number of deaths followed a Poisson distribution. Stata 17.0 (StataCorp LP; College Station, Texas, USA) statistical package was used for the analysis.

## Results

Table I shows the characteristics of the study participants. Only 10% of the patients were men. The mean age of all patients was 77 years (SD 8), ranging from 45 to 96 years. Only 8% smoked but 42% reported alcohol use. The minority had the following chronic diseases: 12% had diabetes, 5% had Alzheimer’s disease and 11% had had a stroke. Self-reported health was excellent in 20% of patients, moderate in 60% and bad in 20%. Previous falls were reported by 76% of patients, and fractures by 71%. Balance difficulties were common (73%) and 44% were unable to walk or had difficulties in walking 500 m.

**Table I.** Characteristics of the multifactorial falls prevention programme patients.

Characteristics	Women *n*=473	Men *n*=54	All *N*=527 [*n*]^a^
Age, mean (SD)	76 (8)	78 (9)	77 (8) [527]
BMI, mean (SD)	26.7 (5.0)	26.8 (4.6)	26.7 (4.9) [494]
Smoker, *n* (%)	32 (7)	6 (13)	38 (8) [493]
Alcohol use, *n* (%)	179 (41)	23 (48)	207 (42) [484]
Diabetes, *n* (%)	53 (11)	12 (22)	65 (12) [527]
Alzheimer’s disease, *n* (%)	23 (5)	3 (6)	26 (5) [527]
Cerebrovascular disease, *n* (%)	48 (10)	11 (20)	59 (11) [527]
SRH, *n* (%)			[490]
Good/excellent	91 (21)	9 (18)	100 (20)
Moderate	264 (60)	29 (59)	293 (60)
Bad	86 (20)	11 (22)	97 (20)
History of falling, *n* (%)	337 (76)	39 (80)	376 (76) [494]
Balance disorder, *n* (%)	316 (71)	46 (90)	362 (73) [494]
Fractures, *n* (%)	323 (73)	28 (57)	351 (71) [493]
Unable or difficulty in walking 500 m, *n* (%)	185 (42)	30 (60)	215 (44) [491]
FTSTS, s, mean (SD)	19.5 (11.4)	20.1 (11.1)	19.5 (11.3) [489]

a Number of those for whom information was available.

BMI: body mass index; SRH: self-reported health; FTSTS: Five Times Sit To Stand.

In the whole case-cohort patients, a total of 2923 person-years were followed up: 254 in men and 2669 in women. The follow-up of the control group included 8153 person-years: 734 in men and 7419 in women.

**Figure 1.**
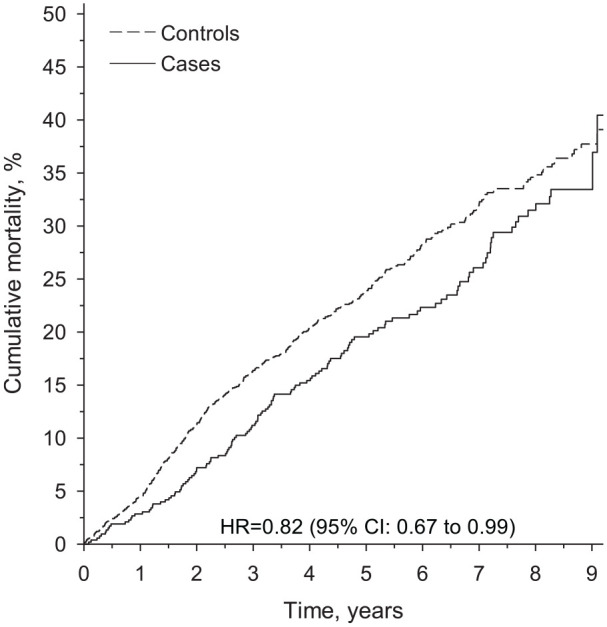
Cumulative all-cause mortality rate. HR: hazard ratio; CI: confidence interval

Altogether, 131 patients (21 men and 110 women) and 447 controls (56 men and 391 women) died during the nine-year follow-up. Figure 1 shows the cumulative all-cause mortality. Hazard ratio was 0.82 (95% CI 0.67 to 0.99), *p*=0.041.

During the nine-year follow-up, the mortality of all patients was 40.4 (95% CI 30.9 to 51.6) and of all controls was 39.1 (95% CI 35.0 to 43.5). Mortality of men among patients was 46.2 (95% CI 31.1 to 64.3) and among controls was 45.4 (95% CI 35.4 to 56.6). Mortality of women among patients was lower (35.8 (95% CI 27.6 to 45.6)) than among controls (38.3 (95% CI 34.0 to 42.9)).

During the five-year follow-up, mortality of all patients was 19.5 (95% CI 16.3 to 23.4) and of all controls was 23.9 (95% CI 21.8 to 26.2). Mortality of men among patients was 34.0 (95% 22.4 to 49.3) and among controls 32.8 (25.5 to 41.5). Mortality of women was lower among patients (17.9 (95% CI 14.7 to 21.9)) than among controls (23.0 (95% CI 20.8 to 25.3)).

**Figure 2.**
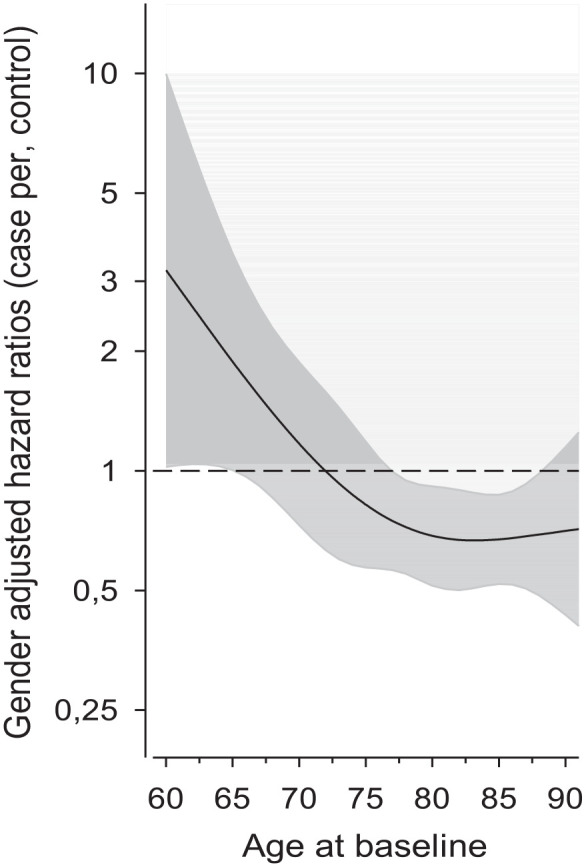
Sex-adjusted hazard ratios of all-cause mortality for cases compared with controls as the function of the age at baseline. Hazard ratios were derived from a three-knot restricted cubic spline model. Shaded area represents 95% confidence intervals [25].

Sex-adjusted hazard ratios between participants and controls according to continuous age at baseline are shown in Figure 2. The MFFP seems to lower the risk for death among patients aged 72 years or older.

Table II shows that compared with controls patients had a 2.69 (95% CI 1.14 to 6.39) times greater risk to die owing to accidents. Instead, patients had lower risk to die owing to dementia (sHR 0.54, 95% CI 0.34 to 0.84).

**Table II.** Sex-adjusted subhazard ratios for most important causes of death.

Causes of death	Cumulative incidence in presence of competing events		sHR^a^ (95% CI)
	Case % (95% CI)	Control % (95% CI)	
Neoplasms C00–D48	5.4 (3.2 to 8.2)	7.0 (5.5 to 8.7)	0.66 (0.41 to 1.06)
Dementias F00–F03	10.6 (8.6 to 12.9)	12.3 (4.9 to 23.5)	0.54 (0.34 to 0.84)
Diseases of the circulatory system I00–I99	16.3 (12.5 to 20.7)	17.2 (13.9 to 21.0)	0.94 (0.71 to 2.29)
External causes of mortality V01–Y98	2.5 (1.2 to 4.7)	0.8 (0.4 to 1.5)	2.69 (1.14 to 6.39)

a Subhazard ratio; competing-risks regression model was used where the rest of the causes of death were considered as competing risks.

CI: confidence interval.

The SMR of patients was 0.85 (95% CI 0.71 to 1.00) and of controls 1.14 (95% CI 1.04 to 1.25), *p*=0.003.

## Discussion

The results show that patients with high risk of falling and osteoporosis and who participated in the MFFP seem to have a lower all-cause mortality compared with the population-based controls matched by age, sex and residence. The population-based controls had not attended the MFFP. The study group had multimorbidity, difficulties in functioning, a possible history of previous falls and, thus, their prognosis was already hindered. Both groups belonged to the same health care system and their health care needs would have been taken care of by the same health care provider.

Although the all-cause mortality rate and the death rates of dementia, neoplasms and diseases of the circulatory system of the case group were lower than the control group’s, the external causes of mortality, which included injuries, was higher. The primary reason for this finding may be that many of the participants had already fallen before or were at high risk of a fall. Second, the participants were still independent enough to participate in the MFFP and move, although with difficulties. Persons with difficult memory disorders were probably not among the study group. The study group also lacked institutionalized older people at baseline. Third, the MFFP might have had an impact on the overall health of the subjects. In their systematic review, Warburton and Bredin discovered that exercise is associated with a reduced risk for all-cause mortality and several chronic medical conditions, for example, cancer and common lifestyle diseases [21]. They discovered that the greatest relative benefits are seen at lower doses of physical activity [21]. This modest activity improvement was also the aim of the MFFP in Lahti [22].

Older patients need individual evidence-based guidance to utilize proper exercise, nutrition, medication and healthy lifestyle choices [2,9–15]. Deprescribing has been reported to provide small reductions in mortality [13], but we found no studies indicating that MFFP could prevent deaths. In their meta-analysis, Beswick et al. found that MFFPs reduced nursing-home admissions, hospitalizations and falls, but not deaths [9]. MFFPs can be utilized to improve physical function and maintain independent living [2,9–15], which is in itself a worthy goal.

The most common causes of death in Finland in order are: 1) diseases of the circulatory system; 2) neoplasms; 3) memory diseases; and 4) accidents, which typically are falls and stumbles [26]. In Finland, two-thirds of fatal falls happened to persons aged over 80 years [26]. The average age of death by falling for Finnish men is 82 years and for women is 88 years [26]. The emergent numbers of falls of the older adults underpins a major health care problem and highlights the need to initiate and continue MFFPs for the prevention of fall-related injuries and premature mortality [1–4]. The Ministry of Social Affairs and Health in Finland has a programme that aims to prevent accidents and deaths caused by accidents [4]. The primary goal is to reduce the number of accidents that lead to loss of health and deaths by 25% by 2030 [4].

The selection of a control group in a cohort study has an important effect on the interpretation of the results [27]. The population-based controls should not have the same disease or the same exposure at baseline as the study group [27]. The controls can be hospital or health care provider-based or population-based. Especially, in retrospective design, several difficulties may emerge when procuring data of the unexposed controls [28].

Our aim was to study the influence of a MFFP on mortality in patients with a risk of osteoporosis and falls. The control group should have had the same risks. Because of the retrospective setting, we were unable to collect such a group. We had to compromise and select population-based controls matched by sex, age and residence, which are usually the most relevant factors for matching [28]. We tried to decrease the selection bias of unknown factors by using three controls per one patient. For further cohort studies we recommend a control group with an elevated risk of osteoporosis and falls from another community that does not have a MFFP.

This retrospective population-based cohort study has many limitations. In real life, you cannot avoid all confounding factors. This is a major disadvantage of the study. It is possible that the study group differed from the population, which is problematic [27]. Few men participated in the MFFP, which might have had an impact on the findings. The register-based data we had about population-based controls also lacked important background factors, such as morbidity and functional ability. These variables are relevant when mortality is assessed. The population-based control group’s age, residence and sex were the only characteristics known. Unfortunately, we were not able to separate fall-related accidents from others, because the patient registers did not have that data. However, the situation is the same in both groups.

The study group might have been more autonomous and their ability to function might have been better than in controls because the patients were able to participate in the MFFP. However, the background factors of the study group showed that they represent patients with at least moderate decrease in SRH and difficulties in walking or balance. The control group might have included institutionalized persons who were not able to participate in the MFFP. In Lahti, the proportion of institutionalized persons was 0.47% of the whole population in 2010 [29]. The MFFP was standardized, thoroughly defined, but it was carried out by only a few health care professionals, whose professional competence might have had an impact on the MFFP’s effectiveness. Every patient had been given an individual training regime, but we do not know how intensively the MFFP patients followed them. All these limitations may influence the generalization of the results.

One strength of the study is that it is a long-term follow-up with sex-, age- and residence-matched controls. All of the MFFP patients participated in the primary treatment formed of the physiotherapist’s and GP’s contacts and, thus, were included in the mortality analyses. This study describes how a MFFP works in real life in relation to lower all-cause mortality. The mortality data derived from the Central Statistical Office of Finland uses a unified and standard methodology and therefore is reliable in covering the whole Finnish population. It ensures that the mortality data is not under-reported. The long length of the follow-up is also a fortifying feature of this study, which ensures that the MFFP is associated with a long-time survival rate.

The population-based controls had not participated in the MFFP. With this study design we can conclude that patients with high risk of falls and osteoporosis that had participated in the MFFP have higher mortality due to external causes such as injuries, but lower all-cause mortality compared with sex-, age- and residence-matched controls who had not participated in the MFFP.

A randomized controlled trial is needed to study the effectiveness of the MFFP on mortality. Further studies should also evaluate the cost-effectiveness of the MFFPs and the effects of the MFFP in other cultures as well.

## Conclusions

The MFFP seems to relate to a lower all-cause mortality when comparing MFFP patients with their age-, sex- and residence-matched controls. However, the MFFP did not seem to relate to a lower injury-related mortality. The relationship between the MFFP and lower all-cause mortality seemed to be strongest in the patients aged 72 years or older. Due to the study setting and population-based control group, it is difficult to draw solid conclusions and further studies are needed. A randomized-controlled trial comparing the MFFP with standard care would give better insight on the effectiveness of a MFFP on mortality.
